# Role of PSMA-ligands imaging in Renal Cell Carcinoma management: current status and future perspectives

**DOI:** 10.1007/s00432-022-03958-7

**Published:** 2022-02-25

**Authors:** Luca Urso, Angelo Castello, Giovanni Christian Rocca, Federica Lancia, Stefano Panareo, Corrado Cittanti, Licia Uccelli, Luigia Florimonte, Massimo Castellani, Carmelo Ippolito, Antonio Frassoldati, Mirco Bartolomei

**Affiliations:** 1 Department of Translational Medicine, University of Ferrara, Via Aldo Moro 8, 44124 Ferrara, Italy; 2 Nuclear Medicine Unit, Oncological Medical and Specialists Department, University Hospital of Ferrara, Ferrara, Italy; 3 Department of Nuclear Medicine, Fondazione IRCCS Ca’ Granda, Ospedale Maggiore Policlinico, Milan, Italy; 4 Urology Unit, Surgical Department, University Hospital of Ferrara, Ferrara, Italy; 5 Oncological Medical and Specialists Department, Oncology Unit, University Hospital of Ferrara, Ferrara, Italy; 6 Nuclear Medicine Unit, Oncology and Haematology Department, University Hospital of Modena, Modena, Italy

**Keywords:** Renal Cell Carcinoma, Prostate-specific membrane antigen, Positron emission tomography, PSMA PET/CT, Clear cell Renal Cell Carcinoma, Renal neoplasms

## Abstract

**Background:**

Renal masses detection is continually increasing worldwide, with Renal Cell Carcinoma (RCC) accounting for approximately 90% of all renal cancers and remaining one of the most aggressive urological malignancies. Despite improvements in cancer management, accurate diagnosis and treatment strategy of RCC by computed tomography (CT) and magnetic resonance imaging (MRI) are still challenging. Prostate-Specific Membrane Antigen (PSMA) is known to be highly expressed on the endothelial cells of the neovasculature of several solid tumors other than prostate cancer, including RCC. In this context, recent preliminary studies reported a promising role for positron emission tomography (PET)/CT with radiolabeled molecules targeting PSMA, in alternative to fluorodeoxyglucose (FDG) in RCC patients.

**Purpose:**

The aim of our review is to provide an updated overview of current evidences and major limitations regarding the use of PSMA PET/CT in RCC.

**Methods:**

A literature search, up to 31 December 2021, was performed using the following electronic databases: PubMed, SCOPUS, Web of Science, and Google Scholar.

**Results:**

The findings of this review suggest that PSMA PET/CT could represent a valid imaging option for diagnosis, staging, and therapy response evaluation in RCC, particularly in clear cell RCC.

**Conclusions:**

Further studies are needed for this “relatively” new imaging modality to consolidate its indications, timing, and practical procedures.

## Introduction

Renal Cell Carcinoma (RCC) is the most common type of renal malignancy, with a worldwide incidence of approximately 400,000 cases per year (Bray et al. 2018; Ahn et al. 2019). In the last 50 years, a progressive increase of RCC diagnosis has been observed, in relation to the exposure to “modern” risk factors (e.g., obesity and alcohol consumption) and to the increased detection by new imaging modalities (Padala et al. 2020; Tung and Sahu 2021). Indeed, kidneys are one of the most frequent sites of incidental findings, of which a non-negligible percentage is diagnosed as malignancies few years later (O’Connor et al. 2012; Ballard and Guzman 2021).

RCC includes two main subtypes, namely clear cell RCC (ccRCC) and non-clear cell RCC (nccRCC), with this latter comprehending a minimum of 15 histotypes, including papillary (pRCC) and chromophobe (chrRCC) (Tung and Sahu 2021). ccRCC, pRCC and chrRCC, respectively, account for 75–80%, 10–15% and 5% of all RCC (Evangelista et al. 2020). As reported by several literature evidences, 20–30% of RCC present with metastatic disease at diagnosis, with a correlated poor prognosis of approximately 10–20% survival rate at 5-year (Ljungberg et al. 2011; Dabestani et al. 2016; Ahn et al. 2019; Tung and Sahu 2021). Currently, partial or radical nephrectomy remains the mainstay of treatment for renal malignancies (Liu 2016).

Imaging modalities of first choice for the characterization of primary RCC are contrast-enhanced computed tomography (ceCT) and magnetic resonance imaging (MRI). On the contrary, [^18^F]-Fluorodeoxyglucose (FDG) Positron Emission Tomography/Computed Tomography (PET/CT) has a limited role in primary RCC evaluation. This is due to the physiological renal excretion of FDG and the expression of different enzymes across RCC subtypes. In particular, the expression of fructose 1,6-bisphosphatase 1, a key enzyme in the gluconeogenesis pathway, appears to be inversely correlated with FDG avidity in ccRCC, resulting with FDG images characterized by low tumor-to-background ratio (Aide et al. 2003; Liu 2016; Chen et al. 2019; Pozzessere et al. 2019).

In the metastatic disease, evaluation by ceCT represents the gold standard imaging modality, while FDG PET/CT is carving out a role in case of inconclusive radiological findings and for treatment surveillance (Aide et al. 2003; Pozzessere et al. 2019). However, several studies reported a potential advantage for FDG PET/CT in comparison to the conventional imaging in RCC restaging, in particular for the detection of early metastatic disease and musculoskeletal metastases, although lesions’ size and low tumor grade can be associated with false-negative results (Park et al. 2009; Wang et al. 2012; Bertagna et al. 2013; Alongi et al. 2015; Liu 2016).

Due to the above-mentioned FDG PET/CT limits, there is a raising interest for the identification of new PET radiotracers able to study RCC. For example, mutations in von Hippel–Lindau (VHL) gene play a primary role in ccRCC pathogenesis, determining a reduction of Hypoxia-inducible factors (HIF) degradation and simulating a hypoxic state (pseudohypoxia). As a result, HIF accumulates and translocates into the cell nucleus where it promotes the transcription of neoangiogenic and growth factors (Chachami et al. 2009; Frew and Moch 2015; Wohlrab et al. 2018; Lopez et al. 2020). This molecular pathway has leading to the discover of hypoxia-related radiotracers, such as [^18^F]-Fluoromisonidazole (FMISO) and [^18^F]-Fluoroazomycin Arabinoside (FAZA) (Carlin et al. 2014). However, these radiotracers did not meet the expectations in RCC. For example, a recent study by Capitanio et al. (Capitanio et al. 2021) assessed the possible role of FAZA PET/CT in the identification of lymph-node metastases in RCC. Nevertheless, no nodal metastasis showed positive FAZA uptake, suggesting that VHL-induced pseudohypoxia phenomenon in RCC is not feasible to be studied with hypoxia-related radiotracers, as it does not represent a real hypoxic state.

Moreover, in the recent years, PET/CT using prostate-specific membrane antigen (PSMA), labeled with ^68^Ga or ^18^F, has revolutionized the imaging of prostate cancer. In fact, PSMA is a transmembrane glycoprotein significantly overexpressed on the epithelial cells of prostate cancers, particularly in those with aggressive biology, high Gleason score, and advanced stages (Bravaccini et al. 2018; Ahn et al. 2019). However, PSMA is also overexpressed on the endothelial cells of the neovasculature of several other solid tumors, including RCC, paving the way to evaluate a possible role of PSMA PET/CT beyond the conventional application in prostate cancer (Chang et al. 2001; Baccala et al. 2007; Demirci et al. 2014; Siva et al. 2020).

In this narrative review, we summarize the current evidences regarding PSMA PET/CT in RCC, highlighting both the usefulness and the limits, and drawing possible future perspectives.

## Methods

A literature search was performed using the following electronic databases: PubMed, SCOPUS, Web of Science, and Google Scholar. The last search was run on 31 December 2021. The following keywords were used: Renal Cell Carcinoma” AND “PSMA PET/CT”, and “renal cancer” AND “PSMA PET/CT”. Only articles in English were selected. Case reports, letters to the editor, and original articles were also included. The references of each article were checked to retrieve any additional paper meeting the inclusion criteria.

### PSMA-ligands imaging

During the last decade, PSMA-ligand PET/CT widespread as a reliable tool in prostate cancer (Ozgül Ekmekcioglu et al. 2019). Several PSMA-ligands have been evaluated over time, labeled with different radionuclides, mainly ^68^Ga and ^18^F.

**Fig. 1:**
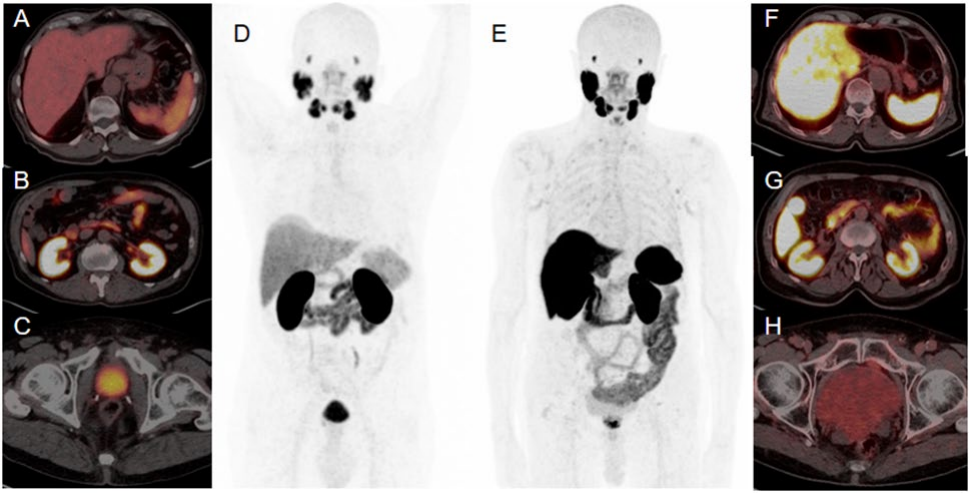
Physiological distribution of [^68^Ga]-PSMA11 PET/CT (**A**–**D**) and [^18^F]-PSMA-1007 PET/CT (**E**–**H**). [^18^F]- PSMA-1007 has a lower urinary clearance in comparison to [^68^Ga]-PSMA11 (**C** vs. **H**), but a higher hepatic elimination (**A** vs. **F**)

[^68^Ga]-PSMA11 is one of the most used radiotracers and the most evaluated in available literature data regarding RCC. It binds to the extracellular domain of the PSMA receptor, being consequently internalized into the cell. Due to its high receptor affinity and to its low molecular weight, [^68^Ga]-PSMA11 demonstrates an excellent tissue penetration into solid lesions, including bone metastases (Afshar-Oromieh et al. 2016). [^68^Ga]-labeled PSMA-ligands are administered intravenously with a recommended activity of 2 MBq/Kg of body weight (Schwarzenboeck et al. 2017). Image acquisition is recommended 1 h after injection, even though some reports described an increased tumor-to-background ratio (TBR) 3 h after injection (Afshar-Oromieh et al. 2016; Schwarzenboeck et al. 2017). PSMA is physiologically expressed in several tissues, such as lacrimal and salivary glands, kidneys, liver and spleen, bowel and bladder (Fig. 1A–D).

[^18^F]-labeled radiotracers addressing PSMA have a great relevance today, with [^18^F]-PSMA-1007 and [^18^F]-DCFPyL recently evaluated also in RCC (Chen et al. 2011; Foley et al. 2020). Indeed, ^18^F has cyclotron production and longer half-life in comparison to ^68^Ga (110 vs. 68 min, respectively), and may therefore allow to ship the radiotracer to a greater number of Nuclear Medicine centers and to perform more daily exams (Foley et al. 2020). Moreover, literature evidences report that [^18^F]-labeled radiotracers addressing PSMA have a lower urinary clearance within the first 2 h after injection in comparison to ^68^Ga ones (Schwarzenboeck et al. 2017). In particular, [^18^F]-labeled radiotracers addressing PSMA are eliminated by the liver and this characteristic may potentially increase the TBR for evaluating renal masses (Giesel et al. 2018; Foley et al. 2020) (Fig. 1E–H). As for [^68^Ga]-PSMA11, [^18^F]-based-PSMA radiotracers’ imaging acquisition is recommended 1 h after 200–250 MBq intravenous injection, with a possible late scan 2–3 h after injection to optimize image interpretation (Chen et al. 2011; Giesel et al. 2018; Foley et al. 2020).

### PSMA PET/CT in RCC diagnosis and staging

Despite an increasing interest in PSMA PET/CT in patients with RCC, the current literature still lacks robust evidence in this context, being confined mainly to single case reports and few reviews (Van De Wiele et al. 2019; Evangelista et al. 2020; Uijen et al. 2021). Moreover, prospective studies are rare; therefore, drawing some generalized conclusions is premature. Current evidences are listed in Table 1.

**Table 1:** Summary of PSMA PET/CT studies in RCC patients

Authors	Number of patients	PSMA-ligand	Study type	Setting	Histology	Comments
Marafi et al. (2020)	1	^18^F-PSMA-1007	Case report	Staging	ccRCC	^18^F-PSMA-1007 PET/CT showed intense of primary renal tumor and bilateral lung nodules
Sawicki et al. (2017)	6	^68^Ga-PSMA-11	Retrospective	Staging	4 ccRCC, 1pRCC, 1 chrRCC	5/6 primary RCC showed PSMA uptake but TBR-SUVmax was low. 8/16 metastases were PSMA positive with a high MBR-SUVmax. All PSMA negative metastasis were subcentimetric lung nodules
Raveenthiran et al. (2019)	38	^68^Ga-PSMA-11	Retrospective	Staging and Restaging	Staging: 8 ccRCC, 1 pRCC, 1 oncocytoma, 6 unknown; Restaging: 20 ccRCC, 1 chrRCC, 1 transitional cell carcinoma	Staging: 16 patients enrolled. 75% showed primary avid lesions. Management was changed for the 43.8%. PSMA PET/CT and ceCT agreed only in the 37.5%. Restaging: 22 patients enrolled. Management was changed in 40.9%. PSMA PET/CT and ceCT agreed only in the 36.4%. Management was changed due to the identification of new sites of suspected metastasis and/or detection of synchronous primaries
Siva et al. (2017)	8	^68^Ga-PSMA-11, ^18^F-FDG	Retrospective	Staging and therapy response evaluation	7 ccRCC, 1 pRCC	PSMA uptake is typically more intense than FDG in RCC. The 2 radiotracers are concordant for detection of sites of disease except in 2 cases (1 of which was papillary carcinoma). ^18^F-FDG PET demonstrated a more rapid response to treatment, but both PET/CT demonstrated responses earlier than the conventional imaging
Tariq et al. (2021)	11	^68^Ga-PSMA-11, ^18^F-FDG	Retrospective	Staging and Restaging	10 ccRCC, 1 unclassified	For primary tumor assessment, ^68^Ga-PSMA-11 and ^18^F-FDG were concordant in 2 patients, and discordant in 3. For metastatic disease, dual tracers were concordant in 9/11 patients. In 3 patients, change was determined a change in clinical management
Saadat et al. (2018)	1	Not reported	Case report	Staging mRCC	ccRCC	^18^F-FDG seems superior to PSMA PET/CT in detecting RCC associated tumor thrombus
Rhee et al. (2016)	10	^68^Ga-PSMA-11	Prospective	Staging	8 ccRCC, 1pRCC, 1 unclassified RCC	PSMA PET/CT detected multiple histologically proven RCC metastases undetected by ceCT, changing patient’s management
Gao et al. (2020)	36	^68^Ga-PSMA-11	Retrospective	Pre-operative	ccRCC	SUVmax could differentiate WHO/ISUP grade (3–4 vs. 1–2), pT stage and adverse pathology (benign vs. malign) with a cut-off value of 16.4
Golan et al. (2021)	27	^68^Ga-PSMA-11	Prospective	Pre-operative	18 ccRCC, 4 pRCC, 2 chrRCC, 2 Oncocytoma, 2 Angiomyolipoma, 1 Mixed Epithelial and Stromal Tumor	29 renal masses were evaluated. Malignant masses (24/29) had median SUVmean and SUVmax significantly higher than benign, a lower wash-out coefficient (K2) and were associated with a positive PSMA staining (20/24)
Rowe et al. (2015)	5	^18^F-DCFPyL	Prospective	Recurrency	ccRCC	Sites of putative metastatic disease were readily identifiable by abnormal 18F-PSMA uptake in each of the 5 patients studied, with overall more lesions detected than on the conventional imaging
Yin et al. (2019)	8	^18^F-DCFPyL	Prospective	Staging/Restaging	nccRCC: 3 pRCC, 2 chrRCC, 2 unclassified RCC, 1 Xp11 traslocation RCC	PSMA PET/CT seems not appropriate to image nccRCC
Meyer et al. (2019)	14	^18^F-DCFPyL	Prospective	Restaging	ccRCC	17/21 (81.0%) metastatic lesions detected on the conventional imaging had radiotracer uptake. 3/3 primary ccRCC had PSMA uptake. In 4 (28.6%) patients, 12 more lesions were identified on PSMA PET/CT than the conventional imaging and 3 of those were no longer considered oligometastatic
Guhne et al. (2021)	9	^68^Ga-PSMA-11	Retrospective	Recurrency	ccRCC	Among 12 PSMA-positive lesions evaluated, 11 were ccRCC metastases and 1 prostate carcinoma at histology. Molecular PSMA expression was detected in all lesions, but intensity and distribution did not correlate with PET parameters (particularly in small lung nodules)
Mittlmeier et al. (2021)	11	^18^F-PSMA-1007	Prospective	Therapy response evaluation	ccRCC	Highly diverging results emerged between PSMA PET/CT and ceCT therapy response evaluation. PSMA PET/CT might allow more precise response assessment to systematic treatment especially for patients showing SD at ceCT
Seront et al. (2021)	2	^68^Ga-PSMA-11	Retrospective	Therapy response evaluation	ccRCC	^68^Ga-PSMA-PET is a promising imaging for early prediction of response to systemic treatment in ccRCC

*18F-FDG* ^18^F-Fluorodeoxyglucose, *ccRCC* clear cell Renal Cell Carcinoma, *ceCT* contrast-enhanced computed tomography, *chrRCC* chromophobe Renal Cell Carcinoma, *MBR* metastasis-to-background, *mRCC* metastatic Renal Cell Carcinoma, *nccRCC* non-clear cell Renal Cell Carcinoma, *PET/CT* positron emission tomography/computed tomography, *pRCC* papillary Renal Cell Carcinoma, *PSMA* prostate-specific membrane antigen, *RCC* Renal Cell Carcinoma, *SD* stable disease, *TBR* tumor-to-background ratio

One of the main limitations for clinical use of PSMA PET/CT in non-metastatic ccRCC is related to the high tracer uptake of normal kidney parenchyma owing to physiological expression in the proximal convoluted tubules and renal excretion of the radiotracer (Siva et al. 2020), although this latter aspect could be overcome with [^18^F]-PSMA radiotracers, which have biliary excretion rather than urinary clearance as [^68^Ga]-PSMA (Giesel et al. 2016; Pianou et al. 2019; Siva et al. 2020). Indeed, in a recent case report, Marafi et al. (Marafi et al. 2020) showed an intense [^18^F]-PSMA concentration in a renal mass, which was proven to be a grade 3 ccRCC. On the other hand, a previous pilot study on the role of [^68^Ga]-PSMA PET/CT in the initial staging demonstrated not significant radiotracer uptake in the primary renal lesions with a mean of TBR-SUVmax of only 0.2 ± 0.3 due to high uptake in the surrounding renal parenchyma (Sawicki et al. 2017). In a recent retrospective study by Raveenthiran and colleagues (Raveenthiran et al. 2019), [^68^Ga]-PSMA PET/CT imaging performed in patients with various RCC histotypes (8 ccRCC, 6 unknown, 1 pRCC, and 1 oncocytoma) showed metastases not identified by standard CT scans in 2 patients. Moreover, PSMA PET/CT did not demonstrate significant uptake in regional lymph nodes of 6 patients that were considered pathologic by standard CT, and identified new synchronous primaries in three cases. Furthermore, patient management was changed in 7 out of 16 RCC patients (43.8%) who performed [^68^Ga]-PSMA/CT for primary staging with respect to the initial stage according to CT features. In addition, Siva et al. (Siva et al. 2017) compared the diagnostic performance of [^68^Ga]-PSMA PET/CT with the conventional CT and FDG PET/CT in a small cohort of 8 RCC patients (7 ccRCC and 1 pRCC). In particular, [^68^Ga]-PSMA and FDG PET findings were concordant in 6 out of 8 patients, although [^68^Ga]-PSMA showed higher uptake in two additional lesions that changed patient’s management from stereotactic radiotherapy to systemic chemotherapy. Likewise, [^68^Ga]-PSMA and FDG PET/CT were mostly concordant for evaluation of primary tumor and metastatic disease in another study with 11 RCC patients (Tariq et al. 2021). Furthermore, dual tracer PET/CT outperformed the conventional imaging in 5 out of 11 patients (45%), detecting more lesions in 2 patients and refuting positive lesions with conventional imaging in another 3 patients. FDG uptake better characterized renal vein thrombus compared to [^68^Ga]-PSMA as also showed in a recent case report, suggesting that friable tumor thrombus with minimal neovasculature may be imaged better with FDG (Saadat et al. 2018).

As we have observed from above-mentioned studies, PSMA PET might have a role in the assessment of lymph-node status and in the detection of occult metastases. Similarly, Rhee and colleagues (Rhee et al. 2016) demonstrated the superiority of [^68^Ga]-PSMA PET/CT able to depict 4/10 regional lymph nodes compared to only 2 classified as metastatic by ceCT. Overall, PSMA PET reported a sensitivity of 92% vs. 67% of ceCT scan with a positive likelihood ratio of 35 and 3, respectively.

More recently, Gao et al. (Gao et al. 2020) investigated the role of [^68^Ga]-PSMA PET/CT for characterizing pathological features of primary tumors in a cohort of 36 ccRCC patients who underwent surgery. SUVmax was significantly different according to histologic grade, pT stage, and adverse pathology (tumor necrosis or sarcomatoid or rhabdoid feature), while Hounsfield values did not show any significant difference. Of note, SUVmax could effectively differentiate WHO/ISUP grade (3–4 vs. 1–2) and adverse pathology (positive vs. negative), with AUC 0.89 (95% CI, 0.81–0.98, *p* < 0.001), cut-off 16.4, sensitivity 100%, and specificity 71% and AUC 0.92 (95% CI, 0.85–0.99, *p* < 0.001), cut-off 18.5, sensitivity 94%, and specificity 87%, respectively. Interestingly, another recent study by Golan and colleagues (Golan et al. 2021) evaluated the diagnostic performance of dynamic [^68^Ga]-PSMA PET/CT in a prospective case series of patients with primary renal mass (18 ccRCC, 4 pRCC, 2 chrRCC, and 5 benign lesions). In their study, the median SUVmean and SUVmax were significantly different between benign and malignant lesions (2.3 vs. 6.8 for SUVmean, respectively, *p* < 0.01; 3.8 vs. 9.4 for SUVmax, respectively, *p* < 0.01). Likewise, the median wash-out coefficient (K2) was significantly lower in malignant lesions than in benign lesions (0.17 vs. 0.70, *p* = 0.02). Increased [^68^Ga]-PSMA tracer uptake and intratumoral retention correlated with PSMA expression in malignant renal tumors compared with benign renal masses, supporting further assessment of dynamic PSMA as a potential tool for evaluating localized renal masses.

Despite growing interest in the clinical use of PSMA PET imaging for improved detection of RCC lesions, some limitations should be mentioned. In particular, small liver lesions below the resolution power of PET/CT (5 mm) as well as small lung metastases, due to free-breathing PET/CT acquisitions, might be overlooked (Ljungberg et al. 2011; O’Connor et al. 2012). However, novel technical improvements might overcome such limitations.

### PSMA PET/CT in RCC recurrence and therapy response evaluation

A few more experiences have been reported in literature regarding the use of PSMA PET for restaging RCC. The first study was reported in 2015 by Rowe et al. (Rowe et al. 2015), who prospectively imaged 5 patients with metastatic ccRCC (mccRCC), comparing [^18^F]-PSMA PET/CT with either ceCT or MRI. Overall, 28 out of 29 lesions were detected by PET/CT, while only 18 were described on conventional imaging. The only lesion that did not show uptake on PSMA PET/CT was a small (6 mm) hepatic lesion, probably undetectable both due to the small size and to the physiological hepatic uptake. On the contrary, the majority of the lesions detected on PSMA PET/CT, but not on conventional imaging, were small mediastinum and retroperitoneum lymph nodes, but also pancreatic, lung, bone and brain metastasis, as well as uncommon sites of disease (paraspinal musculature and subcutaneous soft tissues). SUVmax of detected lesions ranged between 1.6 and 19.3.

In 2017, Siva et al. (Siva et al. 2017) performed a direct comparison between FDG PET and [^68^Ga]-PSMA PET/CT in a cohort of 8 patients with oligometastatic RCC (7 ccRCC and 1 pRCC) before and after treatment, i.e., surgery or stereotactic ablative body radiation. Despite detecting the same sites of disease in 6 patients, PSMA avidity resulted higher than FDG (mean SUVmax 11.4 [5.3–26.5] and 4.4 [3.1–7.0], respectively). However, the two radiotracers showed different response to radiotherapy, with FDG treatment-induced changes coming faster (3–4 months) than on PSMA PET/CT (6–12 months).

The first experience with PSMA PET/CT in nccRCC was published by Yin and colleagues in 2019 (Yin et al. 2019). In their prospective study, the authors imaged 8 patients with metastatic nccRCC (3 pRCC, 2 chrRCC, 1 Xp11 translocation RCC, and 2 unclassified) by [^18^F]-PSMA PET/CT and using either ceCT or MRI as standard of reference. Overall, 10/73 metastatic lesions demonstrated PSMA uptake, while 14 had an equivocal uptake and 49 were defined as non-PSMA avid. In 3 cases, the primary renal tumor was in place, but none of them showed significant PSMA uptake. Therefore, these results suggested that PSMA PET/CT was not appropriate to image nccRCC.

Again, using [^18^F]-PSMA PET/CT, Meyer et al. (Meyer et al. 2019) analysed 14 patients with oligometastatic ccRCC (< 3 lesions on conventional imaging). PET/CT scan detected all 3 renal primary tumors and 29 metastatic localizations, whereas 21 were reported on conventional imaging, represented by CT or MRI. As a consequence, 3 patients were no longer considered oligometastatic after PSMA PET/CT. Only 4 lesions detected on conventional imaging did not show radiotracer uptake (2 retroperitoneal lymph nodes, 1 adrenal gland, and 1 mediastinum localization). The reported detection rates of PSMA PET/CT and conventional imaging were 88.9% and 66.7%, respectively.

Similarly, in 2019, Raveenthiran et al. (Raveenthiran et al. 2019) performed PSMA PET/CT for staging and restaging patients with RCC. Twenty-two out of 38 patients performed [^68^Ga]-PSMA PET/CT for suspected RCC recurrency (20 ccRCC, 1 chrRCC, and 1 transitional cell carcinoma). PSMA PET/CT identified new disease localizations in 9 cases, refuted suspicious lesions in 7 cases, and changed clinical management in 9 patients (40.9%). Moreover, 4 patients had a new synchronous primary neoplasm diagnosis compatible with prostate cancer. Only in 8/22 cases (36%), diagnostic CT and [^68^Ga]-PSMA PET/CT were concordant. Although the study was retrospective and limited by the lack of consistent histological correlation, it suggests a possible utility for [^68^Ga]-PSMA PET/CT in the management of RCC.

More recently, Guhne and colleagues (Gühne et al. 2021) correlated [^68^Ga]-PSMA PET/CT uptake and histopathological findings in 9 patients with suspect metastatic recurrence of ccRCC. Eleven PSMA-positive lesions were confirmed as metastases from RCC, while 1 lesion resulted from prostate cancer. Median SUVmax and SUVmean were 3.1 and 2.0, respectively, with lung metastases showing lower tracer uptakes in comparison to other localizations. No correlation was found between PSMA PET/CT uptake and microvasculature PSMA expression or tumor grade on histopathology.

The first evidence in therapy response assessment was reported by Mittlmeier et al. (2021) in 2021. Eleven patients with mccRCC performed [^18^F]-PSMA PET/CT before starting Tyrosine Kinase Inhibitors (TKI) or Immune Checkpoints Inhibitors (ICI) and after 8 weeks of therapy. Despite all patients were PSMA positive at baseline, 3 patients showed a complete response, 3 a partial response, 4 a stable disease, and 1 a progressive disease at follow-up, according to a modified PERCIST response criteria (Seitz et al. 2018). On the contrary, patient’s responses according to RECIST criteria were 0 complete responses, 1 partial response, 9 stable disease, and 1 progressive disease, respectively. Therefore, the authors concluded suggesting a potential role for [^18^F]-PSMA PET/CT in early therapy response assessment, in particular for patients showing SD at ceCT. Similarly, a recent case report confirmed the potential utility of PSMA PET/CT in the evaluation of response to TKI-ICI therapy in a mccRCC patient (Seront et al. 2021).

## Discussion and future perspectives

The main dilemma in front of incidental renal masses is to distinguish between benign vs. malignant lesions. Nevertheless, in several cases, CT and MRI are unable to reliably distinguish the nature of a renal mass, particularly between avidly enhancing malignant ccRCC and benign oncocytomas (Pierorazio et al. 2013; Pozzessere et al. 2019). As a consequence, a non-negligent percentage of indeterminate findings hesitates in over-treatment and, potentially, in avoidable nephrectomies (Rowe et al. 2017). In this context, the role of molecular imaging for the assessment of renal masses would be of great impact. While FDG PET/CT failed in this subset of patients (Özülker et al. 2011), promising results were reported using ^99^m-Tc-sestamibi SPECT/CT in a few experimental studies (Gormley et al. 1996; Hendrikse et al. 1998; Rowe et al. 2017). In particular, oncocytomas seem to have more mitochondrial content in comparison to ccRCC and may therefore show an increased ^99^m-Tc-sestamibi uptake. Anyhow, patients studied with ^99^m-Tc-sestamibi are a small number and some renal masses are reported to show an intermediate behaviour, in particular chrRCC. Moreover, this imaging is not in the current clinical practice and experimental protocol is needed. Although a relative low number of evidences regarding PSMA PET/CT in RCC are currently available, preliminary results are encouraging both for staging and response assessment, as well as for PSMA-targeted therapy. If preliminary results, such as the higher uptake and the different wash-out kinetics in tumors with more aggressive histologies or pT stage, were confirmed in larger prospective cohorts, PSMA PET/CT would open the way for its clinical routinely use to correctly identify the renal masses that really deserve surgical treatment (Gao et al. 2020; Golan et al. 2021). These papers on the role of molecular imaging with PSMA PET/CT strengthen previous clinico-pathological evidences, such as the study of Spatz et al. (Spatz et al. 2018). This study demonstrated an increased PSMA expression in ccRCC (82.5%) in comparison to other RCC subtypes, and reported higher endothelial PSMA expression in tumor vessels of higher grade and stage, and metastatic and lethal ccRCC. The same Authors also reported a stronger association between PSMA expression and overall survival in comparison to established clinical parameters, highlighting a prognostic relevance of PSMA expression in ccRCC. However, future clinical trials are necessary, to correlate PSMA PET/CT avidity with genetic and biologic tumor characteristics, as well as with radiomics features. The analysis of all these factors could potentially contribute to better stratify patients’ disease, providing a powerful prognostic tool for the oncologists, at least in ccRCC and chrRCC, which seem to be the prevalent PSMA expressing RCC subtypes (Baccala et al. 2007; Spatz et al. 2018; Toyama et al. 2021). On the contrary, PSMA expression is reported to be typically low in pRCC (Baccala et al. 2007; Spatz et al. 2018; Yin et al. 2019), although pRCC is well imaged with FDG PET/CT (Hou et al. 2021).

In staging and restaging setting, ceCT is currently the gold standard, even though false negatives are common in case of small metastatic localizations (Ahn et al. 2019; Pozzessere et al. 2019). FDG PET/CT has demonstrated good sensitivity and specificity (respectively, 86% and 88%) in a meta-analysis by Ma et al. (Ma et al. 2017), despite RCC is not a typical “Warburg” tumor (Lindenberg et al. 2019). Few papers have recently compared FDG and PSMA PET/CT in staging RCC. PSMA-avid lesions seem to show higher uptake compared to those FDG-avid, even though vein thrombus seem to be better detectable on FDG PET/CT (Siva et al. 2017; Saadat et al. 2018). PSMA PET/CT looks particularly promising in detecting oligometastatic disease, in particular small retroperitoneal lymph nodes, which do not reach CT dimensional criteria for disease localizations, changing the therapeutic decision in a non-negligible percentage of patients (Rowe et al. 2015; Rhee et al. 2016; Raveenthiran et al. 2019). Therefore, patients with locally advanced RCC, particularly ccRCC, and uncertain conventional imaging findings could ideally benefit of PSMA PET/CT to better stage their disease and to decide the best treatment option available. Table 2 illustrates ongoing clinical trials with PSMA PET/CT in patients with RCC. Among the reported studies, it is of particular interest a prospective single centre trial (NCT04987086), that is currently recruiting 300 patients for staging locally advanced RCC with [^68^Ga]-PSMA PET/CT, with results available in a couple of years.

**Table 2:** Summary of the ongoing clinical trials with PSMA PET/CT (source: https://clinicaltrials.gov/)

Trial identifier number	Phase	status	PSMA tracer	Aim
NCT04987086	NA	Recruiting	^68^Ga-PSMA-11	(1) To evaluate the diagnostic efficacy of ^68^Ga-PSMA PET in metastatic lesions of locally advanced and advanced RCC, and to compare with that of ceCT. (2) To evaluate whether ^68^Ga-PSMA PET can change the treatment decision of patients with locally advanced and advanced RCC.
NCT05170555	NA	Recruiting	^68^Ga-PSMA-11	(1) To evaluate the uptake of ^68^Ga-PSMA in RCC compared to 18F-FDG. (2) To assess the feasibility of ^177^Lu-EB-PSMA-617 treatment in patients with the advanced RCC.
NCT03427476	I	Completed	^18^F-CTT1057	To test a novel diagnostic PET imaging agent, binding PSMA expressing tumors, for safety and biodistribution.
NCT03387514	II	Completed	^18^F-DCFPyL	To assess response to systemic therapy (anti-angiogenesis and/or immune-based therapies) in patients with mRCC comparing PSMA imaging with the conventional RECIST 1.1 criteria and histopathological endpoints (including isolation, enumeration, and staining of circulating tumor cells).
NCT03073395	I	Recruiting	^68^Ga-P16-093	(1) To evaluate the uptake of ^68^Ga-P16-093 in metastatic prostate and renal cancer. (2) Measurement of the whole-body biodistribution of ^68^Ga-P16-093 in prostate cancer patients to generate human radiation dosimetry data.
NCT04147494	Early I	Recruiting	^68^Ga-PSMA-11, ^68^Ga-FAPI-46	(1) To define the biodistribution of radiotracers in normal and cancer tissues of patients with various non-prostate malignancies, including RCC. (2) To evaluate whether ^68^Ga-PSMA-11 uptake correlates with the amount PSMA in excised cancer tissue.

*ceCT* contrast-enhanced Computed Tomography, *mRCC* metastatic Renal Cell Carcinoma, *NA* not applicable, *PET/CT* positron emission tomography/computed tomography, *PSMA* prostate-specific membrane antigen, *Recist 1.1* Response Evaluation Criteria In Solid Tumors version 1.1., *RCC* Renal Cell Carcinoma

In patients with metastatic disease, PSMA PET/CT has demonstrated a very high detection rate (Rowe et al. 2015; Meyer et al. 2019; Raveenthiran et al. 2019; Gühne et al. 2021). In this subset of patients, a possible future role for PSMA imaging may be in the therapy response evaluation. In oligometastatic disease, stereotactic ablative body radiation is a valid therapeutic option, but preliminary evidences report a slower response on PSMA PET/CT when compared to that detectable with FDG (Siva et al. 2017). Antiangiogenic drugs, such as TKI and Mammalian Target of Rapamycin Inhibitors, as well as Immune Checkpoints Inhibitors, are the standard of care for mRCC systemic treatment (Pichler and Heidegger 2017; Escudier et al. 2019; Pozzessere et al. 2019; Powles et al. 2021). As PSMA is expressed in the neovasculature of RCC, this feature could help in the selection of patients with increased intrarenal tumor-driven angiogenesis, who could potentially benefit from antiangiogenic drugs (Toyama et al. 2021). Moreover, providing an in vivo readout of neovascular density in RCC lesions, PSMA PET/CT could be a valid tool to assess response to treatment and to define the correct timing for stopping systemic therapy (Evangelista et al. 2020). Such a help could be of great utility to identify patients in pseudo-progression after target therapies. This phenomenon consists of a transient increase in tumor volume and represents an important limit of conventional imaging for discriminating it from true progression (Pozzessere et al. 2019). A preliminary work from Mittlmeier et al. (Mittlmeier et al. 2021) provides a good starting point for future works on this perspective, particularly in patients with SD at RECIST 1.1 on ceCT. Future trials should also focus on the analysis of the relationships between PSMA PET/CT changes in therapy response assessment and overall survival.

In mRCC patients with high PSMA avidity, another future perspective is represented by Radioligand Therapy. In a theranostic approach, mRCC could be first imaged with PSMA PET/CT and subsequently treated with PSMA labeled with β-emitting (such as ^177^Lu or ^90^Y) or α-emitting (such as ^225^Ac) radionuclides, as it happens today for neuroendocrine tumors and prostate cancer (Zhang et al. 2021; Uccelli et al. 2021). This approach could be particularly useful in chrRCC, where PSMA expression is usually high and therapeutic options are limited (Baccala et al. 2007; Spatz et al. 2018; Toyama et al. 2021). Moreover, as radioligand therapy is usually a well-tolerated treatment, future evidences are welcome also in patients with suboptimal clinical conditions and limited therapeutic opportunities. Table 3 summarizes current evidences and future opportunities of PSMA-ligands imaging in the new oncological field of RCC.

**Table 3:** Current utility and future perspectives of PSMA-ligands imaging and ^18^F-FDG PET/CT in the different subtypes of RCC (−, +, + + for low, intermediate and high utility of the imaging, respectively; values between brackets represent future perspectives)

Hysthotype	PSMA-ligands Imaging				^18^F-FDG PET/CT		
	Staging	Restaging	Therapy Response assessment	RLT	Staging	Restaging	Therapy Response assessment
ccRCC	+ +	+ + +	+ ( +)	(+ +)	+	+	+
chrRCC	+	+ +	(+ +)	(+)	−	−	−
pRCC	−	−	−	−	+ +	+ +	+ +

*18F-FDG* ^18^F-Fluorodeoxyglucose, *ccRCC* clear cell Renal Cell Carcinoma, *chrRCC* chromophobe Renal Cell Carcinoma, *PET/CT* positron emission tomography/computed tomography, *pRCC* papillary Renal Cell Carcinoma, *PSMA* prostate-specific membrane antigen, *RLT* Radioligand Therapy with radiolabelled PSMA-ligands

The main limitations to this work are represented by the relative low number of RCC patients studied with PSMA PET/CT and the retrospective nature of most of the studies reported. Moreover, RCC comprehends a spectrum of very heterogeneous histotypes, which complicate a unitary and homogeneous analysis of the literature. Finally, the use of different PSMA radiotracers available, some labeled with ^18^F and some with ^68^Ga, represents an additional confounding factor. In fact, despite having multiple radiotracers is a good prospect for the future use of this imaging modality, the lack, at present, of robust papers directly comparing currently available radiotracers undoubtedly represents a limitation in the analysis of literature data.

## Conclusions

This work emerges that PSMA PET/CT has the credentials to represent a valid imaging option in RCC. The main limitations of its current use in clinical practice are the relative low number of patients investigated and the high heterogeneity of RCC. Despite these drawbacks, the premises are encouraging for staging and restaging locally advanced, oligometastatic, and mRCC, in particular ccRCC. Undefined renal masses evaluation and therapy response assessment (TKI, mTOR and ICI in particular) are other promising indications, that should be further explored in the near future. Finally, in a theranostic approach, PSMA-based RLT could represent a future treatment option in mRCC expressing PSMA at PET imaging.

## Data Availability

Not applicable.
